# Meta-analysis of the effects of gamma irradiation on chicken meat and meat product quality

**DOI:** 10.14202/vetworld.2024.1084-1097

**Published:** 2024-05-15

**Authors:** Raissha Rizqi Asmarani, Tri Ujilestari, Muhammad Miftakhus Sholikin, Wulandari Wulandari, Ema Damayanti, Muslih Anwar, Siska Aditya, Mohammad Faiz Karimy, Satriyo Krido Wahono, Endy Triyannanto, Danung Nur Adli, Rio Olympias Sujarwanta, Teguh Wahyono

**Affiliations:** 1 Graduate Student, Animal Science Faculty, Universitas Gadjah Mada, Sleman 55281, Indonesia; 2 Research Center for Food Technology and Processing, National Research and Innovation Agency of Indonesia, Gunungkidul 55861, Indonesia; 3 Research Center for Animal Husbandry, National Research and Innovation Agency of Indonesia, Bogor 16911, Indonesia; 4 Animal Feed and Nutrition Modelling Research Group (AFENUE), IPB University, Bogor 16680, Indonesia; 5 Center for Tropical Animal Studies (CENTRAS), The Institute of Research and Community Empowerment of IPB (LPPM IPB), Bogor 16680, Indonesia; 6 Department of Animal Products Technology, Animal Science Faculty, Universitas Gadjah Mada, Sleman 55281, Indonesia; 7 Department of Feed and Animal Nutrition, Faculty of Animal Science, Universitas Brawijaya, Malang 65145, Indonesia

**Keywords:** chicken, gamma irradiation, meat, meta-analysis, product

## Abstract

**Background and Aim:**

Irradiation is one of the most effective microbial decontamination treatments for eliminating foodborne pathogens and enhancing chicken meat safety. The effect of gamma irradiation on the overall quality of chicken meat and its products must be observed to provide a comprehensive explanation to the public. This meta-analysis examined the effects of gamma irradiation on the oxidation parameters, microbial activity, physicochemical characteristics, sensory parameters, and nutrient quality of chicken meat and meat products.

**Materials and Methods:**

We conducted a literature search using various search engines (Scopus®, PubMed®, and Google Scholar®) with “irradiation,” “gamma,” “chicken,” and “meat” as keywords. Gamma irradiation treatment was set as a fixed effect, and the difference between experiments was set as a random effect. This study used a mixed-model methodology. After evaluation, we selected 43 articles (86 studies) for inclusion in the database.

**Results:**

Gamma irradiation significantly increased (p < 0.01) thiobarbituric acid-reactive substance levels on days 0, 7, and 14 of storage. Gamma irradiation reduced total aerobic bacteria, coliforms, *Salmonella*, yeast, and mold activity (p < 0.01). According to our meta-analysis, 21.75 kGy was the best dose for reducing total aerobic bacteria. On day 0, gamma irradiation did not affect the color parameters (L*, a*, b*). However, a significant difference (p < 0.01) was noted for a* and b* parameters between the control and irradiation treatments at 7 and 14 days. Although irradiation treatment was less consistent in sensory parameters, overall acceptability decreased on days 0, 7, and 14 after storage (p < 0.05). Regarding nutrient composition, gamma irradiation reduced moisture content and free fatty acid (FFA) content (p < 0.05). Although irradiation significantly reduces the microbial population, it increases the oxidation of chicken meat and its products. Irradiation decreases FFA content and overall acceptability, but it does not affect flavor, tenderness, juiciness, or cooking loss.

**Conclusion:**

Gamma irradiation positively reduces the microbial activity in chicken meat and its products but increases the oxidation parameters. Although gamma irradiation does not alter the flavor, tenderness, juiciness, or cooking loss, gamma irradiation can reduce the FFA content and overall acceptability.

## Introduction

In developing countries, poultry meat is an important source of protein due to its low price compared with red meat. In addition, poultry meat is also popular in developed countries because of its health benefits. Globally, chicken meat as the main source of poultry products is projected to account for 41% of total protein derived from meat sources by 2030, an increase of 2% over the baseline period [1]. Poultry and beef meat are expected to represent the largest imports of extra meat into Asia and Africa, where consumption growth will exceed domestic production [1]. Producing and disseminating meat with the longest possible shelf life is a primary challenge for traders [2]. Therefore, the food industry utilizes various food preservation techniques, such as cooling, freezing, evaporation, fermentation, and adding chemical preservatives [3]. Moreover, irradiation has been recognized as an effective method for preventing the spread of diseases and parasites and extending the shelf life of products [4].

In 2003, the FAO recommended the irradiation technique in the Codex Alimentarius, and it has been widely adopted in 50 nations, including the United States, Egypt, China, and most Latin American nations [5]. In the food processing industry, irradiation is commonly used to preserve food. Irradiation is one of the most effective microbial decontamination treatments for eliminating foodborne pathogens and enhancing meat safety [6]. Gamma irradiation can be applied to raw materials because of its high level of effectiveness in reducing the number of germs and its capacity to produce little changes to the natural characteristics of the product [7]. Gamma irradiation is more effective than electron beam irradiation against foodborne pathogens [8]. Irradiation treatment is usually combined with packaging [9–11], cooking [12–14], or the addition of antioxidants [15–17] to minimize the effects of free radical reactions that affect meat quality.

Apart from commercial purposes, gamma irradiation research focuses on developing foods for specialized applications, such as space programs, military operations, and care of geriatric and immunocompromised people [18]. However, consumers’ perception and acceptance of irradiated meat are among the most crucial aspects of adopting irradiation technology in meat production [6]. The effect of gamma irradiation on the overall quality of chicken meat and its products must be observed to provide a comprehensive explanation to the public. Previously, Dimov [2] published a meta-analysis on the impact of gamma irradiation on the lipid oxidative processes exhibited by peroxide value (POV) and thiobarbituric acid-reactive substances (TBARS) in raw chicken meat. A meta-analysis on the impact of gamma irradiation on raw chicken meat quality (color and microbiology) [19] has also been published. Fallah *et al*. [20] reviewed the combined effects of ionizing radiation and bio-based active packaging on the quality of muscle food. However, there is no comprehensive study on the effect of gamma irradiation on the quality of raw chicken meat and its products.

Therefore, this meta-analysis aimed to investigate the effects of gamma irradiation on the oxidation parameters, microbial activity, physicochemical characteristics, sensory parameters, and nutrient quality of chicken meat and meat products.

## Materials and Methods

### Ethical approval

Ethical clearance was not required for the present meta-analysis study. This study was designed according to the Preferred Reporting Items for Systematic Reviews and Meta-Analyses (PRISMA) Protocol.

### Study period and location

The study was conducted from August 2, 2023, to October 31, 2023 at the Research Center for Food Technology and Processing, National Research and Innovation Agency of Indonesia and Faculty of Animal Science, Universitas Gadjah Mada, Indonesia.

### Search strategy

The literature search was performed using Harzing’s Publish or Perish version 8 (Windows GUI Edition). Keywords used were irradiation, gamma radiation, chicken, and meat. The outputs were 1129 articles published between 1998 and 2022 from Scopus®, PubMed®, and Google Scholar®. Only scientific articles were included in the selected literature.

### Selection criteria

**Figure-1:**
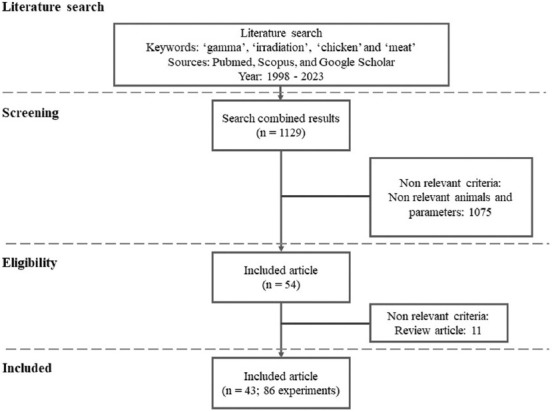
Flow chart for literature selection included in meta-analysis.

The article selection procedure followed the Preferred Reporting Items for Systematic reviews and Meta-Analyses protocol [21]. Inclusion criteria were as follows: (1) The article was published in a scientific journal; (2) the article was an experimental research-based study; (3) the irradiation treatment only uses gamma rays (not electron beams); (4) the irradiation dosage level was reported; and (5) chicken meat or meat products were used as the subjects of this study. Figure-1 shows a diagram of the literature selection. After full-text evaluation, 43 articles (86 experiments) were entered into the database (Table-1) [7, 9–18, 22–53].

**Table 1:** List of eligible studies for meta-analysis of gamma irradiation effects on chicken meat and meat products.

No.	References	Meat sample	Dosage (kGy)	Dosage rate (kGy/h)	Sample treatment
1	Brito *et al*. [7]	Deboned chicken meat	0; and 3	0.32 and 4.04	Untreated
2	Yoon *et al*. [9]	Marinated diced chicken meat	0; 5; 10; 15; 20; 25; and 30	10	Vacuum-packaged
3	Mantilla *et al*. [10]	Chicken breast filets	0; 2; and 3	No information	Vacuum-packaged
4	Nisar *et al*. [11]	Chicken meat	0; 1.5; and 3	No information	Vacuum-packaged and moringa leaf powder addition
5	Jayathilakan *et al*. [12]	Hurdle-processed chicken meat	0; 1; and 2	No information	Cooked with Masala and lactic acid treatment
6	Muhammad *et al*. [13]	Chicken meat	0; 5; and 7.5	No information	Boiled; and fried
7	Chen *et al*. [14]	Chicken dices meat	0; 10; 20	2.1	Stir-fried with dry chili
8	Abdeldaiem [15]	Minced chicken meat	0; 2; 4; and 6	No information	Coating with ethanolic extract of papaya leaves
9	Hwang *et al*. [16]	Chicken sausage	0; 2.5; and 5	5	Untreated; emulsified with mugwort extract; and mugwort extract+ascorbic acid
10	Arshad *et al*. [17]	Chicken meat	0; 1; and 2	No information	Turmeric powder; aerobic packaging; and vacuum packaging
11	Baptista *et al*. [18]	ready-to-eat broiler breast filets	0; and 48	1	Frozen storage
12	Bhoir *et al*. [22]	Minced chicken meat	0; and 0.5	2	Untreated and mixed with Chitosan (0.1% w/w)
13	De Azevedo Gomes *et al*. [23]	Refrigerated mechanically deboned chicken	0; 3; and 4	7.32	Untreated
14	Khalid *et al*. [24]	Chicken meat	0; and 3	No information	Untreated; and treated with 1% and 2% kale leaf powder
15	Javanmard *et al*. [25]	Chicken meat	0; 0.75; 3; and 5	1.98	Frozen storage
16	Rima *et al*. [26]	Fresh chicken meat	0; 1; 2; and 3.5	4.29	Untreated
17	Al-Bachir and Othman [27]	Chicken sausage	0; 2; 4; and 6	8.49	Untreated
18	Hassanzadeh *et al*. [28]	Chicken breast meat	0; and 2.5	No information	Untreated; immersed in chitosan solution; and chitosan solution containing 0.1% grape seed extract
19	Fallah *et al*. [29]	Ready-to-cook Iranian barbecued chicken	0; 1.5; 3; and 4.5	3.06	Untreated
20	Yun *et al*. [30]	Ready-to-eat chicken breast	0; 5; and 40	No information	Heated
21	Mahrour *et al*. [31]	Chicken leg meat	0; and 5	No information	Packed in air and marinated
22	Kim *et al*.[32]	Chicken breast meat	0; 2.5; 5; 7.5; and 10	7	Vacuum-packaged; stored at 5°C and 30°C
23	Zahran [33]	Minced chicken meat	0; 1; and 2	3.49	Untreated; 100; and 200 micro g/g nisin
24	Shankar *et al*. [34]	Boneless chicken thighs	0; and 1	9.57	Untreated and mixed with Essential oils (Oregano+Thyme+Cannelle 2% w/w)
25	Aly and El-Aragi [35]	Cold-sliced chicken meat	0; 2; 4; and 6	1.37	Untreated
26	Mrityunjoy *et al*. [36]	Chicken meat	0; 6; and 8	No information	Untreated
27	Chouliara *et al*. [37]	Chicken breast meat	0; 2; and 4	1	Packed-in air and modified atmosphere packaging
28	Spoto *et al*. [38]	Chicken breast meat	0; 2; and 4	No information	Untreated
29	Irmanita *et al*. [39]	Chicken breast and legs meat	0; 3; and 5	No information	Untreated
30	Al-Bachir *et al*. [40]	Chicken kabab	0; 2; 4; and 6	0.73	Untreated
31	Millar *et al*. [41]	Chicken breast and chicken leg meat	0; and 5	No information	Untreated
32	De Toledo *et al*. [42]	Chicken breast meat	0; 2; 4; 6; and 8	1.2	Untreated; and frozen storage
33	Kang *et al*. [43]	Minced chicken meat	0; 3; 5; 7; and 10	No information	Non-thermal pasteurization
34	Pelicia *et al*. [44]	Chicken breast meat	0; 2; 4; and 8	No information	Vacuum and no-vacuum packaging
35	Kumar *et al*. [45]	Chicken pulav	0; 2; 3; 4; and 5	0.6	Untreated
36	Min *et al*. [46]	Minced chicken meat	0; 1; 3; 5;10; and 15	10	Untreated
37	Kanatt *et al*. [47]	Chicken chilly meat	0; 1; 2; and 3	3	Frozen storage
38	Yusof *et al*. [48]	Chicken sausage and burger	0; 3.5; 5.5; and 10	No information	Untreated
39	Chong-Nam *et al*. [49]	Chicken breast meat	0; 0.5; 1; and 1.5	No information	Cold storage
40	Balamatsia *et al*. [50]	Chicken breast-filet	0; 0.5; 1; and 2	1	Stored Aerobically at 4°C
41	Kanatt *et al*. [51]	Chicken chunks, chicken mince, and chicken leg meat	0; and 2.5	3	Untreated
42	Yoon [52]	Chicken breast meat	0; and 2.55	0.92	Untreated
43	Kanatt *et al*. [53]	Chicken meat	0; and 2.5	2.7	Untreated; treated with citric acid; ascorbic; tocopherol; butylated hydroxytoluene; nitrite; and sodium tripolyphosphate

### Inclusion data

As shown in Table-1, the dosage of gamma irradiation ranged from 0 to 48 kGy and the dosage rate ranged from 0 to 10 kGy/h in this meta-analysis. Meat samples included fresh chicken meat, boneless chicken thighs, minced meat, diced meat, breast filets, marinated meat, chicken sausage, chicken kabab, chicken burger, chicken pulav, chicken chilly meat, and ready-to-cook barbecue chicken. Oxidation parameters were TBARS, total volatile base nitrogen (TVBN), and POV. Myoglobin (Mb), metmyoglobin (MMb), and oxymyoglobin (MbO_2_) were the hemoglobin parameters. Total aerobic bacteria, coliforms, lactic acid bacteria, enterobacteria, *Salmonella*, *Pseudomonas*, staphylococci, yeast, and mold were the microbial load parameters. The pH, lightness (L*), redness (a*), and yellowness (b*) were included physicochemical parameters. The sensory parameters assessed were appearance, texture, taste, color, odor, flavor, tenderness, juiciness, cooking loss, and overall acceptability. Moisture, protein, fat, ash, free fatty acid (FFA) profiles, C14:0, C16:0, C16:1, C18:0, C18:1, C18:2, C18:3, C20:3n6, C20:4, C22:0, saturated fatty acids, monounsaturated fatty acids, and polyunsaturated fatty acids were the nutrient composition.

### Statistical analysis

The database had compatible measurement units and statistical meta-analysis based on mixed-model methodology [54, 55] was performed. Various studies were classified as random effects, and gamma irradiation dose was classified as fixed effects. All statistical analyses were conducted using R software version 4.1.2 by the R Core Team (http://www.r-project.org/index.html) [56] equipped with lme4 library version 1.1-28 (https://cran.r-project.org/web/packages/lme4/index.html). The root-mean-square error (RMSE) and the determination coefficient of Nakagawa or R_GLMM_(c)^2^ were used to validate the model [56–58]. Statistical significance was stated at p < 0.05. When the p-value ranged between 0.05 and 0.10, there was a tendency for the result to be significant.

## Results

The effects of gamma irradiation on the oxidation parameters of chicken meat and its products are summarized in Table-2. Irradiation treatment increased TBARS and POV after 0, 7, and 14 days of storage (p < 0.01). However, gamma irradiation treatment reduced TVBN after 7 and 14 days of storage (p < 0.05). Furthermore, gamma irradiation increased TBARS after 14 days of storage (p < 0.05). In the present meta-analysis, a dose of 3.24 kGy was sufficient to increase the TBARS value. With regard to hemopigment, irradiation treatment decreased Mb levels after 0, 7, and 14 days of storage (p < 0.01). In contrast, MMb levels increased after gamma irradiation (p < 0.05). With the exception of 14 days of storage, gamma irradiation did not affect the MbO_2_ level. Table-3 shows the effects of gamma irradiation on the microbial activity of poultry meat and meat products (log colony forming unit/g). In general, gamma irradiation treatment reduced microbial activity in meat and meat products (i.e., total aerobic bacteria, coliforms, lactic acid bacteria, enterobacteria, *Salmonella*, *Pseudomonas*, *Staphylococcus*, yeast, and mold; p < 0.05).

**Table 2:** Meta-analysis results of gamma irradiation effects on oxidation parameters and haem pigment of chicken meat and meat products.

Response parameter	Unit	n	Intercept	SE Intercept	Slope	SE slope	p-value	RMSE	R^2^
TBARS (d)	mg MDA/kg								
0		106	0.62	0.09	0.04	0.01	0.001	0.31	0.72
7		76	0.72	0.18	0.17	0.05	0.003	0.56	0.58
14		84	0.78	0.15	0.10	0.02	0.023	0.23	0.88
21		6	0.43	0.23	0.49	0.13	0.129	0.32	0.00
TVBN (d)	mg/100 mL								
0		49	7.02	1.75	0.18	0.06	0.006	1.82	0.90
7		30	9.42	2.42	−0.77	0.27	0.011	1.47	0.94
14		26	11.8	3.58	−1.62	0.67	0.028	3.58	0.84
POV (d)	meq peroxide/kg								
0		38	0.43	0.07	0.08	0.01	0.001	0.08	0.89
7		30	0.52	0.09	0.06	0.01	0.001	0.03	0.99
14		30	0.60	0.09	0.06	0.01	0.001	0.03	0.99
Mb (d)	%								
0		18	36.5	1.44	−0.90	0.22	0.002	0.76	0.93
7		18	22.5	1.13	−0.85	0.20	0.002	0.71	0.91
14		18	11.9	1.85	−0.68	1.19	0.005	0.66	0.97
MMb (d)	%								
0		24	46.7	3.55	0.51	0.18	0.013	0.72	0.99
7		18	49.6	0.98	0.71	0.17	0.002	0.61	0.91
14		18	58.2	1.12	1.07	0.19	0.001	0.69	0.92
MbO_2_ (d)	%								
0		12	12.3	0.62	0.37	0.19	0.108	0.44	0.82
7		12	15.4	0.63	0.33	0.19	0.130	0.43	0.82
14		12	20.4	0.65	0.33	0.13	0.036	0.28	0.93

TBARS=Thiobarbituric acid-reactive substances, TVBN=Total volatile base nitrogen, POV=Peroxide value, Mb=Myoglobin, MMb: Metmyoglobin, MbO2=Oxymyoglobin, SE=Standard error, RMSE=Root mean square error, R^2^=The proportion of a dependent variable’s variation that can be explained by an independent variable (bigger is better)

**Table 3:** Meta-analysis results of gamma irradiation effects on microbial activity of chicken meat and meat products (log colony forming unit/g).

Response parameter	n	Intercept	SE Intercept	Slope	SE slope	p-value	RMSE	R^2^
Total aerobic bacteria (d)								
0	167	4.70	0.44	−0.29	0.07	0.001	4.36	0.14
7	69	5.37	0.28	−0.52	0.07	0.001	1.85	0.45
14	69	7.51	0.28	−0.99	0.09	0.001	1.49	0.69
Coliforms (d)								
0	106	3.86	0.34	−0.58	0.07	0.001	1.55	0.60
7	43	3.09	0.41	−0.49	0.10	0.001	1.29	0.56
14	37	3.74	0.43	−0.63	0.10	0.001	1.31	0.64
Lactic acid bacteria (d)								
0	16	3.87	0.77	−0.60	0.24	0.026	1.34	0.51
7	21	4.63	0.51	−0.99	0.15	0.001	0.79	0.80
14	20	6.30	0.67	−0.95	0.20	0.001	1.32	0.64
Enterobacteriaceae (d)								
0	12	2.07	0.55	−0.51	0.19	0.024	1.10	0.42
7	17	3.98	0.60	−1.19	0.25	0.001	1.54	0.01
14	18	5.02	0.66	−1.29	0.24	0.001	1.75	0.01
Yeast and mold (d)								
0	47	4.32	0.77	−0.54	0.06	0.001	0.75	0.93
7	30	3.83	0.50	−0.78	0.14	0.001	1.42	0.62
14	23	4.45	0.41	−0.76	0.13	0.001	1.39	0.01
*Salmonella* (0 d)	17	5.89	0.82	−0.40	0.09	0.001	0.99	0.74
*Pseudomonas* (d)								
0	12	7.11	0.22	−0.41	0.03	0.001	0.29	0.95
7	9	4.50	0.80	−1.35	0.31	0.003	1.34	0.01
14	6	6.15	1.03	−1.74	0.39	0.012	1.30	0.01
Staphylococcal (d)								
0	37	5.94	0.75	−0.67	0.09	0.001	0.99	0.87
7	7	5.22	1.39	−1.66	0.19	0.003	0.37	0.97
14	5	5.83	1.53	−1.59	0.23	0.018	0.43	0.96

SE=Standard error, RMSE: Root mean square error, R^2^=The proportion of a dependent variable’s variation that can be explained by an independent variable (bigger is better)

Table-4 shows the effects of gamma irradiation on the physicochemical and sensory parameters of meat and products. In terms of physicochemical parameters, gamma irradiation did not have any significant effect on the pH value at 0, 7, and 14 days after storage. Furthermore, on day 0, irradiation did not affect L*, a*, and b* values in meat. In contrast, a* and b* values were elevated after irradiation treatment at 7 and 14 days of storage (p < 0.01). Irradiation treatment decreased the appearance, texture, taste, and odor on storage day 0 (p = 0.01). Overall acceptability at 0, 7, and 14 days of storage decreased with increasing irradiation dose (p < 0.05). A dose of 12.26 kGy was sufficient to decrease the overall acceptability score. Gamma irradiation did not affect flavor, tenderness, juiciness, and cooking loss of meat and its products. Gamma irradiation treatment did not affect the appearance, texture, taste, color, and odor after 7 days of storage. Gamma irradiation did not affect the overall sensory parameters of meat and its product after 14 days of storage, except for taste and acceptability values, which were negatively affected (p < 0.05).

**Table 4:** Meta-analysis results of gamma irradiation effects on physicochemical and sensory parameters of chicken meat and meat products.

Response parameter	n	Intercept	SE intercept	Slope	SE slope	p-value	RMSE	R^2^
pH (d)								
0	59	5.89	0.08	0.01	0.01	0.514	0.07	0.94
7	20	5.92	0.07	0.01	0.01	0.596	0.14	0.37
14	8	5.76	0.05	0.01	0.01	0.789	0.06	0.16
Lightness (L*) (d)								
0	38	53.3	1.59	−0.14	0.11	0.221	0.97	0.96
7	33	52.7	1.38	0.01	0.13	0.999	0.77	0.96
14	27	51.8	1.34	−0.36	0.17	0.048	0.89	0.93
Redness (a*) (d)								
0	38	11.9	1.38	0.11	0.07	0.125	0.58	0.98
7	33	10.3	1.35	0.29	0.07	0.001	0.43	0.99
14	27	10.5	1.30	0.36	0.07	0.001	0.38	0.99
Yellowness (b*) (d)								
0	38	11.6	1.23	0.001	0.06	0.897	0.53	0.98
7	33	10.4	1.02	0.23	0.06	0.001	0.39	0.98
14	27	10.8	0.76	0.41	0.08	0.001	0.46	0.94
Appearance (d)								
0	61	7.83	0.24	−0.04	0.09	0.001	0.29	0.89
7	30	7.64	0.26	−0.04	0.02	0.113	0.14	0.96
14	30	7.34	0.30	−0.02	0.04	0.714	0.27	0.88
Texture (d)								
0	67	7.28	0.23	−0.03	0.01	0.002	0.37	0.83
7	29	6.85	0.37	−0.01	0.02	0.520	0.20	0.95
14	29	6.75	0.37	−0.01	0.016	0.591	0.19	0.96
Taste (d)								
0	60	7.09	0.21	−0.03	0.01	0.001	0.29	0.86
7	35	6.02	0.44	−0.01	0.03	0.689	0.20	0.97
14	26	6.66	0.41	−0.07	0.02	0.001	0.89	0.99
Color (d)								
0	63	7.27	0.31	−0.01	0.01	0.552	0.37	0.91
7	26	7.84	0.57	0.01	0.01	0.729	0.15	0.99
14	26	7.79	0.56	0.01	0.01	0.491	0.18	0.98
Odor (d)								
0	52	7.76	0.23	−0.05	0.01	0.001	0.41	0.79
7	44	6.94	0.39	0.01	0.07	0.890	0.58	0.79
14	44	6.34	0.56	0.09	0.08	0.230	0.63	0.87
Flavor (0 d)	39	7.06	0.49	−0.01	0.02	0.751	0.50	0.87
Tenderness (0 d)	14	5.10	0.26	−0.02	0.03	0.565	0.28	0.60
Juiciness (0 d)	14	5.89	0.23	−0.01	0.04	0.828	0.35	0.29
Cooking loss (0 d)	16	23.5	1.04	−0.02	0.13	0.862	1.28	0.57
Overall acceptability (d)								
0	86	7.41	0.19	−0.03	0.01	0.004	0.50	0.71
7	40	7.57	0.20	−0.02	0.01	0.025	0.13	0.95
14	40	7.54	0.21	−0.02	0.01	0.026	0.14	0.95

SE=Standard error, RMSE=Root mean square error, R^2^=The proportion of a dependent variable’s variation that can be explained by an independent variable (bigger is better)

**Figure-2:**
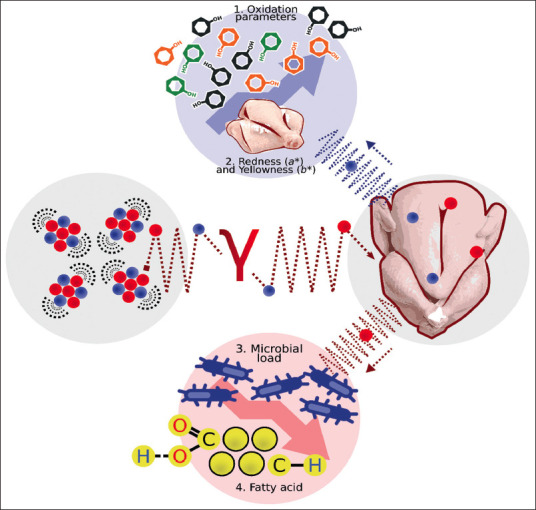
Gamma irradiation influences the oxidation parameters, heme pigment, microbial load, and fatty acid profiles of chicken meat and meat products.

The effects of gamma irradiation on the nutrient and fatty acid characteristics of chicken meat and meat products are summarized in Table-5. Gamma irradiation decreased the moisture and FFA content (7 and 14 days of storage) of chicken meat and its product (p < 0.05). Interestingly, gamma irradiation enhanced crude protein (CP) content (p < 0.01). Meanwhile, irradiation did not significantly affect the fat content, ash, and FFA (0-day storage). Except for C20:3n6 and C22:0, gamma irradiation decreased the fatty acid profile of meat and its products (p < 0.01). Figure-2 shows an illustrative outline of the effect of gamma irradiation on the quality of chicken meat and its products.

**Table 5:** Meta-analysis results of gamma irradiation effects on nutrient and fatty acid characteristics of chicken meat and meat products.

Response parameter	Unit	n	Intercept	SE Intercept	Slope	SE slope	p-value	RMSE	R^2^
Moisture	%	18	71.8	1.84	−0.05	0.02	0.039	0.62	0.97
Protein	%	17	22.6	1.67	0.06	0.02	0.008	0.58	0.97
Fat	%	18	4.46	1.20	0.01	0.01	0.945	0.37	0.97
Ash	%	18	2.65	0.75	−0.01	0.01	0.642	0.14	0.99
Free fatty acids	mg/g meat								
0		18	0.47	0.09	−0.05	0.04	0.293	0.24	0.01
7		14	1.33	0.21	−0.27	0.11	0.049	0.45	0.38
14		14	2.39	0.43	−0.54	0.24	0.047	1.06	0.01
Fatty acids	% Total FA								
C14:0		12	10.6	0.23	−0.08	0.03	0.022	0.09	0.94
C16:0		15	52.8	7.81	−1.15	0.20	0.001	0.69	1.00
C16:1		12	15.3	0.55	−0.30	0.05	0.001	0.17	0.97
C18:0		15	19.8	1.84	−1.11	0.17	0.001	0.59	0.97
C18:1		15	26.9	2.31	−0.87	0.20	0.001	0.69	0.97
C18:2		15	13.1	1.36	−0.35	0.08	0.002	0.27	0.99
C18:3		15	2.25	0.34	−0.17	0.04	0.003	0.15	0.94
C20:3n6		12	0.66	0.12	−0.01	0.01	0.701	0.05	0.93
C20:4		15	2.61	0.54	−0.11	0.03	0.006	0.11	0.99
C22:0		12	3.61	2.73	−0.13	0.11	0.273	0.37	0.99
SFA		12	0.44	0.16	−0.27	0.04	0.001	0.13	0.87
MUFA		12	27.4	0.21	−0.78	0.09	0.001	0.32	0.88
PUFA		12	15.7	0.12	−0.38	0.04	0.001	0.15	0.89

FA=Fatty acids, SFA=Saturated fatty acids, MUFA=Monounsaturated fatty acids, PUFA=Polyunsaturated fatty acids, SE=Standard error, RMSE=Root mean square error, R^2^=The proportion of a dependent variable’s variation that can be explained by an independent variable (bigger is better)

## Discussion

### Influence of gamma irradiation on oxidation parameters of chicken meat and its products

TBARS is one of the oxidation parameters in meat in units of malondialdehyde (MDA). Lipid peroxidation generates MDA as a byproduct. This MDA interacts with thiobarbituric acid to produce pink chromogen TBARS [59]. We measured lipid peroxidation and its secondary products in terms of TBARS [22]. Because sensory evaluation of oxidized odor levels in meat correlates well with TBARS results, the TBARS variable is particularly essential [23, 60]. In the present meta-analysis, irradiation increased TBARS values at 0, 7, and 14 days of storage (p < 0.05). Although this study integrated data from the addition of antioxidants, packaging, and cooking, statistical analysis revealed an increase in the TBARS value. In a previous study, Lee and Ahn [61] demonstrated that irradiation only enhanced TBARS in raw and cooked meat under aerobic packaging conditions, whereas lipid oxidation in irradiated meat did not occur in the absence of oxygen. According to our findings, there is an increase in TBARS in irradiated meat or meat products after additional treatment. Increased gamma-ray dose promoted the production of hydroperoxide. On the other hand, package type contributed to the variability and overall effect of TBARS generation [2]. According to Arshad *et al*. [17], irradiation can increase TBARS in both cooked and raw beef only when packaging is aerobically performed. As the storage time progressed, TBARS levels increased, and the rate of lipid oxidation was higher in irradiated samples than in non-irradiated samples [16]. Khalid *et al*. [24] reported that increasing the irradiation dose enhances the TBARS value. In this meta-analysis, a dose of 3.24 kGy was sufficient to increase the TBARS level. According to Dimov [2], 4 kGy irradiation has no negative effect on raw chicken meat, maintains low oxidation levels, and may be recommended for use in practice.

TVBN may be used as a quality index for chicken meat because its increase is associated with the activity of spoilage bacteria and endogenous enzymes [15]. Non-protein nitrogenous substances and protein breakdown produce TVBN values [24]. Intrinsic factors, such as the amount and types of spoilage microflora and pH, may influence TVBN levels during storage [20], depending on the type of muscle food. In our findings, TVBN increased after radiation treatment at the 0-day storage point. Khalid *et al*. [24] demonstrated that ionization enhances the number of volatile compounds in ready-to-eat chicken breast. TVBN decreased after radiation treatment at the 7- and 14-day storage points. This result is similar to that reported by Arshad *et al*. [17], who observed that TVBN increased on day 0 without radiation treatment but decreased during the 40-day storage interval for irradiated samples compared with non-irradiated samples. This reduction indicates a decrease in spoilage inside the sample, which is a positive indication. Increasing the dose decreases the rate of TVBN synthesis during storage by decreasing the initial concentrations of the most common spoilage bacteria [25].

In addition to TBARS, POV is a crucial quality indicator in irradiated meat samples because it reflects the extent of lipid damage induced by irradiation [11]. Irradiation enhances the lipid oxidation process, which is particularly significant in products with high fat and unsaturated fatty acid content because it generates numerous free radicals [26]. Similar to TBARS values, POV increased with increasing irradiation dose and storage period [17]. Increased gamma-ray dose promoted the production of hydroperoxide [2]. At the end of storage, POV production increased, and the highest POV value was detected at the highest dose of irradiation [24]. Because free radicals created during food irradiation processing are catalysts of lipidic auto-oxidation, a significant increase in POV is consistent with the predictions [11]. In previous studies by Nisar *et al*. [11], Khalid *et al*. [24], the addition of antioxidants prevented lipid oxidation. However, according to our findings, an increase in POV still occurs in post-irradiated samples.

The decrease in Mb level and the increase in MMb and MbO_2_ levels demonstrate that oxidation increases while the gamma irradiation dose increases. Different mechanisms are responsible for the pro-oxidant capacity of Mb. One of these mechanisms is the ability to decompose hydroperoxide, and another is the conversion to ferryl/perferryl form, which allows them to serve as free radicals. Both of these mechanisms are responsible for the pro-oxidant capacity of Mb [17]. With the passage of time and an increase in dose through an intermediary MbO_2_ phase, Mb is oxidized into MMb [62]. MMb-containing meat subjected to irradiation resulted in MbO_2_ regeneration. MMb reacts with free radicals during MbO_2_ regeneration. A small amount of MbO_2_ was produced when purified MMb was irradiated in an aqueous solution [63]. The red color of the meat will change with an increase in both MbO_2_ and MMb. MMb reacts with hydroxyl radicals to generate MbO_2_, resulting in irradiated light meat with a bright red color [6]. However, the change in the Mb level after irradiation also varied according to the meat type. Zhou *et al*. [5] showed that poultry muscles are not drastically discolored because they contain less Mb.

### Influence of gamma irradiation on chicken meat microbial activity and its products

In recent years, there has been a growth in the consumption of foods derived from animals; however, little is known about their contamination with the most prevalent non-spore-producing pathogenic bacteria in foods [8]. The production of chicken-based products at the factory may have been contaminated with a high population of microorganisms, as well as the chicken meat and ingredients used to produce products, which may have contained significant amounts of bacteria [27]. Therefore, gamma irradiation plays an important role in preventing the growth of pathogens in meat. However, the type and quantity of microorganisms present in food affect the effectiveness of the irradiation [28]. In conclusion, gamma irradiation treatment significantly reduced all microorganism activity in chicken meat and meat products. The microbial populations decreased significantly as the gamma irradiation dose increased [17]. Furthermore, based on our meta-analysis, the optimum dose of gamma irradiation was 21.74, 6.84, 3.76, 3.93, 7.82, 7.52, and 8.87 kGy, which removed total aerobic bacteria, coliforms, lactic acid bacteria, Enterobacteriaceae, yeast and mold, *Salmonella*, and staphylococcal activity, respectively.

All types of ionizing radiation inactivate microorganisms through two major mechanisms: Direct interaction with cell components and indirect action from radiolytic products such as H^+^, OH^-^, and e^-25^ [64]. The direct target of ionizing radiation is chromosomal DNA, which loses its function when exposed to radiation. Ionizing water molecules generate reactive hydroxyl radicals that can damage the DNA of microbes, cause base alteration, break the DNA strand, and ultimately result in the death of microbial cells [20]. Arshad *et al*. [17] demonstrated that the total aerobic bacteria and coliform population was decontaminated after irradiation at a dose of 2 kGy in both aerobic and vacuum packaging samples. Irradiation dosages of 1.5 and 3 kGy reduced the initial population of anaerobic mesophilic bacteria by 2 and 3.4 log units, respectively, whereas 4.5 kGy dropped the population below the technique limit of detection during storage [29]. The total aerobic bacteria population decreased due to irradiation treatment has also been observed in red meat, ostrich, and seafood samples [20, 24]. Due to the observed microbial development in the samples irradiated at 5 kGy, specific-purpose foods must be irradiated at a high dose (40kGy) [30]. A combination of gamma irradiation with natural plant extracts [31], antioxidant treatment [24], vacuum packaging [17, 32], bioactive packaging [20], bacteriocin treatment [33], and essential oil treatment [34] is effective in reducing the total aerobic bacteria activity.

Coliform bacteria, which are responsible for spoilage of meat, are present in meat and its products [24]. Coliforms are microorganisms that include both pathogenic and nonpathogenic bacteria [22]. Li *et al*. [65] discovered that total coliforms were more sensitive to irradiation and that treatment with 2 kGy inhibited coliform growth. Increased irradiation intensity has a greater impact on the inactivation of microorganisms in food. In addition, irradiation was used to preserve meat for several days [59]. A previous meta-analysis by Dimov and Popova [19] also showed that irradiated treatment had a reduction effect on the coliform population. Irradiation at 2 kGy reduced coliform counts by approximately 4 log cycles [27]. In addition, coliforms were not detected in samples irradiated at 4 and 6 kGy throughout storage. Irradiation at 4 kGy significantly decontaminated and improved the hygienic quality of chicken-fermented products [66]. Ahn *et al*. [6] reported that the populations of the most common enteric pathogens such as *Escherichia coli*, *Staphylococcus*, and *Salmonella* can be significantly eliminated by a low dose (<3 kGy). As the gamma irradiation dose increases, the *E. coli* viable count decreases [8]. Intrinsic and extrinsic factors affect the intensity of radiation-induced damage, the amount and type of damage, and the radiosensitivity of microbes [20]. Irradiation and frozen storage are more effective in reducing coliforms than either method alone [19]. Irradiation and vacuum packaging are also more effective in reducing coliforms [17]. Reduction in the quantity of coliforms due to the combination treatment (irradiation and other treatments) is a crucial factor contributing to food safety [22]. The radiosensitivity of coliforms changes depending on the isolation source, strain, temperature, irradiation, oxygen content, and food matrix [8].

Among the microbial flora present in chicken meat products, lactic acid bacteria are the most resistant to irradiation treatment [29]. However, Abdeldaiem [15] reported that irradiated samples at doses of 0, 2, 4, and 6 kGy inhibited the development of lactic acid bacteria after 9, 18, 24, and 30 days of storage, respectively. Furthermore, gamma irradiation decreased lactic acid bacteria from day 0 of storage without a combination of treatments [22]. Although lactic acid bacteria are the most ionizing radiation-resistant bacteria, Enterobacteriaceae are the most sensitive bacteria [20]. Another study reported that compared to Enterobacteriaceae and *Pseudomonas*, lactic acid bacteria and *Brochothrix thermosphacta* are more resistant to irradiation [5, 29]. A previous meat study by Chouliara *et al*. [66] showed that irradiating meat at 2–5 kGy is more effective than eradicating lactic acid bacteria in eliminating Enterobacteriaceae and staphylococci.

Yeast and mold counts have been employed as sanitation indicators, and their high levels increase spoilage [33]. Yeasts are the most resistant microbes, followed by lactic acid bacteria, and their decrease in clippings is dose-dependent [66]. Because of their complex genetic structure, yeasts and molds are sensitive to irradiation [29]. However, irradiation should be combined with other treatments to maximize the efficacy of lowering yeast and mold activity. Abdeldaiem [15] reported reduced yeast and mold counts in samples of minced chicken thighs coated with an edible coating containing 2% ethanolic extract of papaya leaves. However, a single irradiation treatment is sufficient to remove yeast and mold. As reported by Aly and Aragi [35], gamma irradiation at doses of 2, 4, and 6 kGy reduced the initial counts of total bacteria, psychrophilic bacteria, spore-forming bacteria, total molds, and yeasts. However, only small volumes of bacterial and fungal growth were detected at 6 and 8 kGy [36]. Jayathilakan *et al*. [12] reported that a 2 log reduction in yeasts and molds can be achieved by applying a dosage of 2 kGy. The application of irradiation to certain chicken products requires further consideration. Meat yeasts, primarily *Debaryomyces* spp. and *Micrococcaceae*, play a secondary positive role in fermented sausages and are widely employed as starter cultures [66].

*E. coli*, *Salmonella* spp., and *Staphylococcus* spp. were highly prevalent in meat samples, which could degrade meat quality and increase the risk of foodborne infections [36]. *Pseudomonas* spp. is Gram-negative microbes regarded as one of the primary meat-spoiling microbes [37]. Mrityunjoy *et al*. [36] reported that *Salmonella* spp., *Shigella* spp., and *Listeria* spp. were completely eradicated (100%) from raw broiler meat following 6 kGy irradiation. Spoto *et al*. [38] reported that a minimum dose of 3.0 kGy is required to protect consumers against foodborne diseases associated with *Staphylococcus aureus*. When every living organism absorbs ionizing radiation during the sterilization process of *S. aureus*, there is a chance that the radiation will strike directly on the DNA, resulting in cell death [39]. Similar to the summary of our findings, irradiation is commonly used in the food processing industry to preserve food goods. It is effective against *E. coli*, *Staphylococcus*, *Salmonella*, and other harmful microorganisms [59].

### Influence of gamma irradiation on the physicochemical and sensory parameters of chicken meat

In our study, gamma irradiation did not significantly affect pH. Similarly, studies by Mantilla *et al*. [10], Yun *et al*. [30], Al-Bachir and Othman [27], Hwang *et al*. [16], and Hassanzadeh *et al*. [28] showed that gamma irradiation had no effect on the pH values of chicken meat and its products during storage. This result may be due to undetected bacterial growth, which may prevent the synthesis of alkaline chemicals and maintain steady pH levels during storage [18]. However, irradiation may change the pH value under special conditions and treatments. Hassanzadeh *et al*. [28] showed that a combination of irradiation and chitosan treatment can decrease pH. Yun *et al*. [30] also reported that the pH of ready-to-eat meat samples irradiated at 5 kGy was not significantly different from that of meat samples irradiated at 40 kGy but was statistically significant. Bachir *et al*. [40] investigated the effect of irradiation on the pH of chicken kebabs and found an increase in the pH following irradiation. This may be due to changes in the chemical properties of the herbs used in the irradiated chicken product. However, further studies are required. In general, pH of aqueous system can influence the irradiation results. An acidic medium (excess H^+^) encourages the loss of aqueous electrons (), whereas an alkaline medium encourages their production [30]. In an extracellular environment in which microorganisms are suspended, pH significantly affects their survival after irradiation [6].

Irradiation alters meat flavor, color, and oxidative alterations, which significantly impact customer acceptance [6]. In this meta-analysis, we summarized color, expressed as total color difference (E), and hue angle (H°, 90° = yellow, 180° = green, and 0° = red) values [16]. On day 0, irradiation did not affect the values of L*, a*, and b* in meat. However, a* and b* values increased after irradiation treatment after 7 and 14 days of storage (p = 0.01). Brito *et al*. [7] also demonstrated a statistically significant difference (p = 0.05) in color parameters between the irradiated and control samples on certain days of refrigerated storage. Variable b* (yellowness) is primarily responsible for the color variation because chicken meat is predominantly yellow [24]. Persistent red pigments or brown pigments that turn red over time appear to result from the binding of irradiation-generated reactive oxygen species (O_2_) or gasses (CO) that form complexes bound by iron under modified reducing conditions [67]. Because of the sensitivity of the Mb molecule, specifically iron, to chemical transformations and changes in the energy input that eventually alter its structure, the irradiated meat changed color during refrigerated storage [7]. When the central iron atom in the heme group of Mb is oxidized, the ferrous heme iron changes to its ferric form, resulting in the transformation from Mb to MMb, which is responsible for meat discoloration [6, 19]. Color changes in irradiated fresh meat are caused by the inherent susceptibility of Mb molecules to energy input and changes in the chemical environment; heme iron is particularly vulnerable [5]. However, hemoglobin levels are higher than Mb levels in the muscles of low-pigmented meats such as chicken meat [41]. Several variables, such as irradiation dose, animal species, muscle type, additives, and packaging type, affect the color variations of irradiated meat [6]. With regard to meat type (i.e., pork meat, chicken meat, or products) and irradiation dose, packaging of meat (oxygen availability) may affect color changes of meat and products following gamma irradiation [68].

Even if there is a benefit to stability, consumers must once again rely on the esthetic appearance of the product in purchasing decisions for most processed poultry offered today. Moreover, consumers are interested in meat characteristics such as texture, flavor, juiciness, and appearance [69]. According to our findings, gamma irradiation affects the majority of sensory parameters (appearance, texture, taste, and odor) only on day 0 of storage or immediately after irradiation. In contrast, no significant effect was observed in the 7^th^ and 14^th^ storages. This pattern is similar to that reported in a previous study by Yoon *et al*. [9], Khalid *et al*. [24]. This shows that the effect of gamma irradiation on sensory parameters is not particularly noticeable after a long storage period. However, further research is required. Interestingly, the overall acceptability at 0, 7, and 14 days of storage decreased after irradiation. This result may be related to the effect of lipid oxidation on chicken meat and products after irradiation. Fats begin to oxidize on their own, resulting in rancid off-flavors when exposed to radiation [70]. Regarding overall quality and aroma, lipid oxidation was not the only major issue because the panelists did not recognize it [71]. Sulfur compounds appear to be the main volatile elements responsible for the odor of irradiated meat [32]. The volatile components responsible for these off-odors appear to result from the effects of electromagnetic energy (gamma radiation, accelerated electrons) on the production of high-energy species that destroy proteins and lipid molecules [72]. Sensorial analysis showed that irradiated samples had a significantly stronger rancid flavor than the control [17, 73]. It has been shown that the use of irradiation can result in some undesired changes in food if treated at high irradiation doses, mostly observed in food such as meat whose color and lipids are the significant indication elements, and a minor change in color and lipids may lead to rejection by consumers [59, 70]. On the other hand, Arvanitoyannis and Stratakos [71] recommended a maximum dose of 10 kGy for food irradiation without the need for toxicological or nutritional tests. These concentrations do not affect the flavor of the food. Variations in the effect of gamma irradiation on sensory parameters can be explained by various factors. Variations in free radicals, color changes, lipid oxidation, and off-odors depend on the irradiation dose and the type of meat [70, 74]. Accordingly, this study can be used as a reference for industries to anticipate changes in the sensory parameters of meat products following irradiation treatment.

In the current study, gamma irradiation did not significantly change tenderness, juiciness, and cooking loss of meat and its products. Therefore, our findings indicate that gamma irradiation does not affect the quality of meat for subsequent processing. The juiciness and tenderness of the irradiated, cooked chicken were only minimally affected and the sensory panel considered it satisfactory [75]. No differences in tenderness and juiciness evaluations were observed between treatments for beef patties irradiated at 0, 3, and 4.5 kGy [71]. Conversely, De Toledo *et al*. [42] reported that irradiation changed the tenderness and juiciness of fresh meat because of liquid loss after irradiation treatment. Rima *et al*. [26] have also demonstrated that the breakdown of myofibrillar and structural proteins after irradiation could reduce the cooking loss of irradiated meat samples. This is important because the disruption of muscle fibers is typically associated with a tenderizing effect [75]. Rababah *et al*. [76] reported that irradiation reduces tenderness in consumers and instrumental evaluations. In addition, infusion of plant extracts enhanced tenderness. These differences may be related to variations in the moisture content of the samples. The lack of variation in shear force and collagen solubility in the meat samples was another factor contributing to these inconsistent results. In addition, we assume that variations in the protein composition of the samples influence the irradiation effect. Proteolysis and protein oxidation play a significant role in the development of tenderness in irradiated meat during storage [77]. Kanatt *et al*. [78] also demonstrated a direct correlation between decreased shear force and increased collagen solubility in buffalo meat samples. In general, the perception of juiciness is increased by increasing moisture and/or fat content [69].

### Influence of gamma irradiation on nutrient and fatty acid characteristics of chicken meat and its products

In conclusion, gamma irradiation decreases the moisture content of chicken meat and its products. This may be due to gamma-ray-induced degradation of meat protein fraction hydration capacity [18]. At higher irradiation dosages, the water content is generally released as a drip and remains on the surface of foods, reducing the moisture content of the food [79]. Chouliara *et al*. [66] observed that irradiation may have changed the functioning of meat proteins in such a way that the water-binding ability decreased, resulting in increased water loss. Moisture content can also be attributed to irradiation-induced protein denaturation [80]. Moisture reduction has a positive impact on the reduction of microbial activity. A lower moisture content helped to extend the shelf life of meat, which may be due to the lower availability of water for microorganisms [81]. However, it also changes the value of juiciness [69].

Based on the results of this study, gamma irradiation alters the protein content of chicken meat and its products. Higher doses of irradiation slightly enhanced the proportion of protein in the irradiated samples [26, 27]. The solubility of collagen and protein in irradiated meat is enhanced [78]. We assume that this change is generated by two aspects: (1) The oxidation of structural proteins in the meat and/or (2) the impact of lipid oxidation, which generates free radicals that interact with the protein. However, this method is also dependent on the presence of other ingredients in the sample, in particular chicken ready-to-eat products. Reactive oxygen species produced by lipid oxidation can change many intracellular and membrane proteins in the muscle [82]. Interactions between free radicals and other dietary components, such as amino acids, lipids, and proteins, are the main source of changes in product quality [68, 70]. Fallah *et al*. [20] demonstrated that radiation processing of muscle meals significantly increased the initial protein carbonyl content by 55.7% (R* = 1.557). In addition, irradiation significantly increased the protein carbonyl content of muscle meals by 17.1% during storage. Irradiation generates alkanes and alkenes that appear to originate from unsaturated fatty acids and amino acids [72]. Changes in food characteristics such as pH, ionic strength, dissolved gas level, viscosity, oxidation-reduction potential, and surface tension can also lead to alterations in enzymatic activity and protein denaturation [5].

In general, gamma irradiation decreases the fatty acid profile of meat and its products. Fatty acid composition plays an important role in determining the nutritional value, flavor, and textural characteristics of meat [62]. Fatty acid profile is typically used to predict lipid degradation in raw meat after irradiation [81]. A decrease in fatty acid content is induced by lipid oxidation. Unsaturated fatty acids are the principal resource for lipid oxidation [72, 83]. Ionizing radiation destroys biological components such as DNA, pigments, fatty acids, and membrane lipids [6]. Unsaturated fatty acids and carbonyl groups (fatty acids and amino acids) have electron-poor carbon-carbon double bonds that are extremely vulnerable to damage caused by free radical [72]. Lipid oxidation not only lowers the nutritional content of muscle meals through the decomposition of essential fatty acids and vitamins but also affects the sensory quality of the products due to the creation of chemicals that generate rancid odor and flavor [20]. Chicken meat is very sensitive to deterioration from oxidation processes because of the presence of relatively large levels of unsaturated fatty acids [81].

## Conclusion

This study presents an in-depth overview of the influence of gamma irradiation on preservation technology in the chicken meat industry. Our results show that gamma irradiation positively reduces the microbial activity in chicken meat and its products but increases the oxidation parameters. Gamma irradiation can reduce the FFA content and overall acceptability but does not alter the flavor, tenderness, juiciness, or cooking loss. Gamma irradiation remains an important method for preserving chicken meat and its products. However, there is a need for a strategy to reduce the effect of oxidation, which affects reducing several sensory parameters in post-irradiated meats.

## Authors’ Contributions

TW, MMS, ED, and ET: Conceptualization. RRA, TW, MMS, ED, DNA, and SA: Investigation. RRA, TW, MMS, TU, and WW: Methodology and formal analysis. RRA, ROS, ED, MA, and SA: Validation. RRA, TW, TU, MFK, and ROS: Data curation and writing of the original draft. RRA, SKW, DNA, MA, ET, and ROS: Resources and writing review and editing. RRA, TW, WW, MFK, SKW, and ET: Revised and edited the manuscript. TW, ET, and ROS: Supervision. All authors have read, reviewed, and approved the final manuscript.
